# Functional antigen presenting cells generated from human dendritic cells (DC) derived induced pluripotent stem cell lines

**DOI:** 10.1186/2051-1426-3-S2-P206

**Published:** 2015-11-04

**Authors:** Arvind Chhabra

**Affiliations:** 1 University of Connecticut Health Center, Farmington, CT, USA

## Background

Human induced pluripotent stem cells (iPS) represent a unique source to create donor specific cells of choice.

## Methods

We here report the generation of induced pluripotent stem cell (iPS) lines from human dendritic cells (DC), through a non-integrating RNA virus, sendai virus, expressing the reprogramming factor genes.

## Results

We show that among the CD4, CD8, and the monocyte populations derived from human peripheral blood derived leukocytes (PBL), monocyte derived DC consistently yield iPS lines than CD4 and CD8 T cells. We here show data from three iPS lines derived from the DC of two different individuals, an under 50 years old younger donor and an over 70 years old older donor. Detailed characterization of these iPS lines show that the lines exhibit normal karyotype and pluripotency phenotype, generated embryoid bodies (EB) that could yield hematopoietic stem cells (HSC) precursors that produced different blood lineage progenitors in a colony forming unit (CFU) assay, and effector T cells in an OP9-Delta-1 feeder based system. We also show that these DC-iPS lines can be re-differentiated into antigen presenting cells (APC), and these iPS-DC can efficiently present human melanoma associated antigenic epitope, MART-127-35, to antigen specific T cells.

## Conclusions

Human DC derived iPS generated APC offer a unique resource to characterize their immunogenic potential in comparison with the peripheral blood derived APC, and also to study developmental process leading to generation of human APC.

